# The Glutamate–Glutamine Axis in Pediatric Septic Shock: Immunometabolic Mechanisms, Biomarker Potential, and Clinical Implications

**DOI:** 10.3390/ijms27114708

**Published:** 2026-05-23

**Authors:** Yaru Cui, Juan Wang, Yiyao Bao

**Affiliations:** Department of Surgical Intensive Care Unit, Children’s Hospital, National Clinical Research Center for Children and Adolescents’ Health and Diseases, Zhejiang University School of Medicine, Hangzhou 310052, China; 6514118@zju.edu.cn (Y.C.); 6513146@zju.edu.cn (J.W.)

**Keywords:** pediatric septic shock, glutamate–glutamine axis, immunometabolism, biomarkers, metabolic reprogramming, prognosis, critical care

## Abstract

Pediatric septic shock remains a major cause of morbidity and mortality in critically ill children and is increasingly recognized as a syndrome of profound immunometabolic dysregulation. This narrative review synthesizes current clinical, translational, and mechanistic evidence on the glutamate–glutamine axis in pediatric septic shock. The review focuses on how glutamine and glutamate metabolism may interact with immune-cell function, mitochondrial substrate handling, redox defense, and intestinal barrier integrity, while distinguishing biological plausibility from validated clinical utility. Current evidence supports the glutamate–glutamine axis as a mechanistically relevant pathway and a source of candidate biomarkers, but pediatric-specific data remain limited and do not yet justify routine biomarker use or glutamine-based intervention in unselected children with septic shock. Future studies should use standardized sampling, reproducible analytical methods, pediatric validation cohorts, and phenotype-guided trial designs before this axis can be translated into clinical decision making.

## 1. Introduction 

Pediatric septic shock remains a major cause of critical illness, multiple organ dysfunction, and death worldwide despite continuing advances in antimicrobial therapy, hemodynamic support, and pediatric intensive care. The updated international consensus criteria published in 2024 further clarified that pediatric septic shock should be understood as a distinct syndrome of infection-associated organ dysfunction with cardiovascular failure rather than as a simple extrapolation of adult sepsis frameworks [1]. This distinction matters because the epidemiology, developmental physiology, host immune response, and clinical trajectories of septic shock in children differ substantially from those of adults. Global epidemiologic data also emphasize the ongoing magnitude of the problem, with pediatric and neonatal sepsis continuing to account for a considerable burden of mortality, particularly in resource-limited settings [2].

A major reason outcomes remain difficult to predict is that pediatric septic shock is not solely an infectious or inflammatory disorder; it is also an immunometabolic disease. Sepsis-associated organ dysfunction emerges from the interaction of pathogen burden, host immune activation, endothelial injury, mitochondrial stress, altered substrate utilization, and maladaptive recovery programs. Contemporary pediatric studies show that immune dysregulation in sepsis includes not only hyperinflammation but also immune suppression, leukocyte dysfunction, altered cell fate, and phase-dependent remodeling of circulating immune-cell populations [3,4]. These observations have shifted attention away from single inflammatory mediators toward integrated host-response networks, among which intermediary metabolism is especially attractive because it sits at the interface of energy production, biosynthesis, redox control, and immune-cell function.

The concept of sepsis immunometabolism has therefore gained prominence as a framework for understanding why children with apparently similar infections can diverge so markedly in disease severity and outcome. During sepsis, metabolic programs are reorganized at both cellular and systemic levels: aerobic metabolism becomes inefficient, mitochondrial function is perturbed, oxidative phosphorylation is compromised, and immune cells adopt altered fuel preferences to sustain activation, effector signaling, or survival under stress [5]. Amino acid metabolism is a particularly important component of this adaptation. Among amino acids, glutamine has long been recognized as conditionally essential during catabolic illness, trauma, and infection, whereas glutamate serves as a central intermediate linking nitrogen handling, anaplerosis, and antioxidant defense [6,7].

Together, glutamine and glutamate form a coordinated metabolic axis regulated by glutaminase, glutamine synthetase, glutamate dehydrogenase, aminotransferases, and amino acid transport systems. This axis links nitrogen trafficking, tricarboxylic acid cycle anaplerosis, nucleotide biosynthesis, ammonia handling, glutathione synthesis, and nutrient-sensitive stress signaling [7,8]. Its relevance to septic shock is therefore not based on a single pathway, but on the convergence of immune-cell substrate demand, mitochondrial fuel use, and redox defense during systemic inflammation [6,7,8].

This axis may be especially important in children. Pediatric patients have ongoing growth demands, developmentally specific substrate handling, and less metabolic reserve than adults in the face of severe systemic inflammation. At the same time, direct pediatric evidence is beginning to suggest that amino acid metabolism is clinically informative in septic shock. Metabolomics studies have shown that serum metabolic profiles can distinguish pediatric septic shock from control states and may be associated with survival, with amino acid pathways repeatedly emerging among the most informative signatures [9,10,11]. Parallel biomarker reviews have emphasized the need for markers that move beyond conventional inflammation indices toward phase-specific and organ-specific biology [12].

At the same time, the translational picture is complex. In mixed critically ill populations, abnormal plasma glutamine concentrations at ICU admission have been associated with adverse outcomes, but both low and high levels may carry risk, suggesting that glutamine behaves as a context-dependent biomarker rather than a linear indicator of disease severity [13,14]. Experimental sepsis work also indicates that glutamine metabolism becomes tissue-specific rather than uniformly suppressed during critical illness [15]. Against this background, the present review examines the glutamate–glutamine axis as a pediatric immunometabolic framework for septic shock, with emphasis on its biologic basis, pathophysiologic relevance, biomarker potential, and therapeutic implications.

### Narrative Review Approach, Literature Selection, and Evidence Appraisal

This article was designed as a narrative review rather than a systematic review or meta-analysis. Accordingly, the manuscript was structured to follow the principles of high-quality narrative review reporting, including a clearly defined topic, explicit literature-search approach, balanced synthesis of supportive and conflicting evidence, critical appraisal of the literature, and conclusions that remain proportionate to the available evidence. PRISMA flow-diagram reporting and quantitative evidence pooling were not applied because the aim was to integrate heterogeneous pediatric clinical data, adult critical-care evidence, experimental sepsis models, metabolomics studies, and the mechanistic literature rather than to answer a single narrowly defined intervention or diagnostic-accuracy question.

To make the search process reproducible, the relevant literature was identified from PubMed, Web of Science, and Google Scholar for records available from database inception to April 2026. The core search strategy used the following concept blocks: (sepsis OR septic shock) AND (pediatric OR paediatric OR child OR children OR infant OR neonatal) AND (glutamine OR glutamate OR glutamate-glutamine axis OR glutamine-glutamate axis OR immunometabolism OR metabolomics OR biomarker OR mitochondrial dysfunction OR intestinal barrier OR critical illness). Searches were supplemented by screening reference lists of highly relevant reviews, clinical studies, and mechanistic articles. Pediatric clinical studies were prioritized when available; adult critical-care studies, animal models, and cell-based studies were included only when they clarified mechanisms, methods, or translational limitations that were not adequately addressed in pediatric cohorts.

Studies were considered eligible when they addressed at least one of the following domains: glutamine or glutamate biology, amino acid metabolism in sepsis or critical illness, pediatric septic shock metabolomics, biomarker development, mitochondrial or redox mechanisms, intestinal barrier or gut–liver axis injury, nutritional support, or metabolic intervention. Articles were excluded when they were unrelated to sepsis or critical illness, focused exclusively on non-sepsis conditions without mechanistic relevance, lacked accessible full text, provided only conference abstracts without sufficient methodological detail, or repeated data already reported in a more complete publication. Because the field includes heterogeneous evidence types, selection emphasized relevance, biological plausibility, pediatric applicability, methodological transparency, and consistency with the purpose of this review.

Evidence was appraised qualitatively with attention to risk-of-bias domains appropriate to each study type. For observational clinical studies, the main considerations were cohort definition, confounding by severity of illness or nutritional status, timing of sample collection, completeness of outcome data, assay validity, and adjustment for competing predictors. For interventional studies and meta-analyses, attention was paid to patient selection, route and timing of supplementation, baseline glutamine status, organ dysfunction, safety signals, and consistency of outcomes. For experimental studies, emphasis was placed on model relevance, biological plausibility, and whether findings were directly transferable to pediatric septic shock. These considerations were used to grade the strength of statements throughout the review as mechanistic, hypothesis-generating, candidate translational, or clinically validated.

## 2. Biological Basis of the Glutamate–Glutamine Axis in Pediatric Septic Shock

### 2.1. Core Components and Physiological Functions of the Glutamate–Glutamine Axis

The glutamate–glutamine axis is a highly integrated metabolic network that coordinates nitrogen transport, carbon flux, cellular bioenergetics, redox balance, and biosynthetic activity across multiple organs. Rather than representing a simple reversible conversion between two amino acids, this axis functions as a dynamic inter-organ and intracellular system linking skeletal muscle, liver, intestine, kidney, brain, and immune cells [16,17,18]. Under physiologic conditions, glutamine is the most abundant free amino acid in plasma and intracellular pools, whereas glutamate serves as a central metabolic intermediate connecting amino acid catabolism, transamination reactions, and entry into the tricarboxylic acid cycle through alpha-ketoglutarate [7,16].

At the biochemical level, the axis is organized around glutamine synthetase, glutaminase, and glutamate dehydrogenase. Glutamine synthetase catalyzes the ATP-dependent conversion of glutamate and ammonia into glutamine and is therefore a key enzyme for ammonia detoxification and glutamine synthesis. Glutaminase hydrolyzes glutamine to glutamate and ammonia, thereby initiating glutaminolysis and making both carbon and nitrogen available for downstream metabolism. Glutamate dehydrogenase then catalyzes the reversible conversion of glutamate to alpha-ketoglutarate and ammonia, linking amino acid turnover directly to mitochondrial oxidative metabolism [19,20,21]. In addition, aminotransferases broaden the metabolic reach of glutamate by connecting it to other amino acid pools and to cellular nitrogen shuttling [17,20,22].

The physiologic behavior of this axis is also determined by membrane transport systems. Glutamine transport is mediated primarily by members of the SLC1, SLC7, and SLC38 families, including ASCT2 and several sodium-coupled neutral amino acid transporters. These transporters do not simply move substrate across the membrane; they also regulate nutrient sensing, amino acid exchange, and intracellular substrate availability [23,24,25]. In immune and epithelial cells, transporter activity helps determine whether glutamine is used for energy production, nucleotide synthesis, or signaling pathways related to growth and stress adaptation [24,25].

One of the most fundamental physiologic functions of the axis is the maintenance of inter-organ nitrogen homeostasis. Skeletal muscle is a major site of glutamine and alanine storage, synthesis, and release; together, these amino acids constitute a large fraction of the free amino acid pool mobilized from muscle during catabolic stress and provide key nitrogen and carbon substrates for gluconeogenesis, ureagenesis, immune-cell metabolism, and acute stress responses. In this setting, muscle-derived glutamine serves as a reservoir that buffers systemic amino acid demand, while the intestine and liver are major consumers under postabsorptive and inflammatory conditions [26,27]. In the liver, glutamine metabolism is spatially organized: periportal hepatocytes preferentially catabolize glutamine for ureagenesis, whereas perivenous hepatocytes express high glutamine synthetase activity to recapture residual ammonia and synthesize glutamine [19]. The kidney likewise uses glutamine catabolism to support ammoniagenesis and bicarbonate generation, linking the axis to acid–base regulation during systemic stress [19,27].

A second major function of the glutamate–glutamine axis is support of cellular energy metabolism and biosynthesis. Once imported into the cell, glutamine can be converted to glutamate and then to alpha-ketoglutarate, replenishing the tricarboxylic acid cycle and sustaining ATP generation. This role is especially important in rapidly proliferating or metabolically active cells, including enterocytes, lymphocytes, macrophages, fibroblasts, and renal tubular cells [28,29,30]. In addition to its energetic role, glutamine provides nitrogen for purine and pyrimidine synthesis and supports nutrient-sensitive signaling pathways such as mTOR [31,32].

The axis is equally central to immune physiology. Immune cells consume glutamine at high rates, in some settings comparable to or greater than glucose utilization, particularly during activation and proliferation [6,7,28]. Glutamine supports lymphocyte clonal expansion, cytokine production, macrophage phagocytic activity, neutrophil microbial killing, and monocyte function by providing both metabolic fuel and biosynthetic substrate [6,7]. This dependence explains why disruption of the axis may affect immune competence far beyond a simple shortage of calories or nitrogen.

Another major physiologic function of the glutamate–glutamine axis is preservation of intestinal structure and function. The small intestine is one of the body’s largest glutamine-consuming organs, and enterocytes preferentially oxidize glutamine as a major respiratory fuel [29,33]. Beyond energy provision, glutamine supports nucleotide synthesis within enterocytes, promotes mucosal growth and repair, helps maintain tight-junction integrity, and contributes to barrier defense against luminal pathogens and toxins [31,33].

Finally, the glutamate–glutamine axis is a major determinant of redox homeostasis. Glutamate is an obligate precursor for glutathione synthesis, and glutamine contributes indirectly by serving as a source of intracellular glutamate. Through this pathway, glutamine availability influences antioxidant capacity, resistance to reactive oxygen species, and mitochondrial stability [7,8]. Taken together, these physiologic roles explain why this axis is biologically well positioned to influence the response to pediatric septic shock.

### 2.2. Dysregulation of the Glutamate–Glutamine Axis in Pediatric Septic Shock

In pediatric septic shock, the physiologic balance of the glutamate–glutamine axis is disrupted by the combined effects of systemic inflammation, hypercatabolism, mitochondrial stress, and altered inter-organ substrate trafficking. Amino acid homeostasis is no longer directed primarily toward growth and tissue maintenance but instead toward stress adaptation, immune activation, gluconeogenesis, and redox defense [34]. Under these conditions, glutamine may shift from an abundant circulating amino acid to a functionally limited substrate when skeletal muscle release is insufficient to match the increased requirements of the immune system, intestinal epithelium, liver, and other metabolically active tissues [15].

Available pediatric data support this view. In critically ill children, plasma glutamine deficiency at admission has been associated with a higher burden of organ failure [35]. Glutamine depletion has also been documented in children with severe meningococcal disease, further suggesting that acute pediatric inflammatory critical illness is accompanied by rapid exhaustion of key amino acid pools [36].

**Figure 1:**
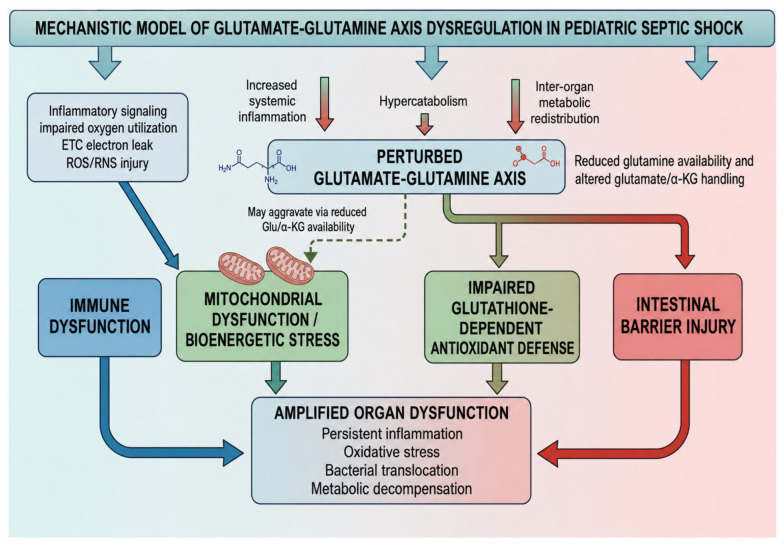
Mechanistic model of glutamate–glutamine axis dysregulation in pediatric septic shock.

These abnormalities are unlikely to be uniform across all tissues or stages of illness. Longitudinal pediatric data indicate that amino acid kinetics and bioenergetic profiles change substantially over the course of severe sepsis and systemic inflammatory response syndrome [37], while adult sepsis studies show accelerated amino acid clearance and redistribution even when total-body amino acid turnover is increased [38]. Accordingly, the glutamate–glutamine axis in septic shock should be interpreted as a dynamic metabolic program rather than a static biochemical abnormality, as shown in Figure 1.

This figure illustrates how severe infection and host-response dysregulation perturb the glutamate–glutamine axis in pediatric septic shock. Increased systemic inflammation, hypercatabolism, and inter-organ metabolic redistribution reduce effective glutamine availability and alter glutamate handling. The model should not be interpreted as implying that altered glutamine or glutamate availability is the primary initiating cause of mitochondrial dysfunction. Rather, inflammatory signaling, impaired oxygen utilization, electron-transport-chain leak, and reactive oxygen and nitrogen species are major upstream drivers of mitochondrial injury in septic shock. Reduced glutamate and alpha-ketoglutarate availability may secondarily limit tricarboxylic-acid-cycle anaplerosis, reducing-equivalent flux, and redox buffering, thereby aggravating immune dysfunction, antioxidant failure, intestinal barrier injury, and organ dysfunction.

### 2.3. Relationship of the Glutamate–Glutamine Axis to Immune Regulation, Energy Metabolism, and Oxidative Stress

The physiologic significance of the glutamate–glutamine axis in pediatric septic shock lies in its connection to three processes that frequently fail during severe infection: immune regulation, cellular energy adaptation, and antioxidant defense. Immune cells require metabolic substrates to sustain proliferation, cytokine production, and antimicrobial activity, while parenchymal cells must preserve mitochondrial function and redox balance under systemic stress [7,34]. Disruption of this axis may therefore contribute to organ injury by weakening several linked adaptive responses rather than by acting through a single linear mechanism.

From an immunologic perspective, glutamine is a critical substrate for lymphocyte activation and proliferation. Mechanistic studies have shown that glutamine availability directly influences T-cell proliferation and cytokine production [39,40]. Glutamine-related amino acid sensing also contributes to mTOR signaling, linking nutrient sufficiency to cell growth, protein synthesis, and immune-cell activation [32].

The same axis is also relevant to energy metabolism, although its role should be interpreted within the broader mitochondrial injury program of septic shock. Through conversion of glutamine to glutamate and then to alpha-ketoglutarate, cells can replenish tricarboxylic acid cycle intermediates and support mitochondrial ATP production when demand is high or glucose utilization is impaired [7,17]. However, in septic shock, mitochondrial dysfunction is more likely initiated by inflammatory signaling, impaired oxygen utilization, electron-transport-chain disturbance, and reactive oxygen and nitrogen species injury. Altered glutamate or alpha-ketoglutarate availability may therefore worsen bioenergetic failure by limiting anaplerotic input and reducing-equivalent flux rather than acting as the sole or primary cause of mitochondrial injury [41].

In parallel, the glutamate–glutamine axis is a major determinant of redox homeostasis. Glutamate is an essential precursor for glutathione synthesis, and glutamine serves as an upstream source of intracellular glutamate [8]. Experimental studies have shown that glutamine can attenuate oxidative stress and apoptosis in injured epithelial tissues [42]. These observations support the view that the glutamate–glutamine axis is not merely a background metabolic pathway, but a central regulator of the balance between host defense, energy adaptation, and oxidative protection, as shown in Table 1.

## 3. Pathophysiological Role of the Glutamate–Glutamine Axis

### 3.1. Effects on Inflammatory Responses and Immune Dysfunction

The glutamate–glutamine axis plays a central role in shaping the inflammatory response during pediatric septic shock because it directly influences the metabolic fitness of both innate and adaptive immune cells. In sepsis, immune dysregulation includes not only excessive inflammation but also impaired pathogen clearance, lymphocyte dysfunction, altered macrophage phenotypes, and later immunosuppression [3,4]. Since glutamine is a major substrate for proliferating lymphocytes and activated macrophages, disruption of its availability can affect both the magnitude and the quality of the host response [7,34].

In innate immunity, glutamine availability is closely linked to macrophage function. Early experimental work showed that glutamine deprivation reduces macrophage phagocytosis, RNA synthesis, and interleukin-1 production [43]. More recent mechanistic studies demonstrate that glutamine supports macrophage metabolic reprogramming during activation and contributes to the remodeling required for inflammatory polarization and resolution-associated programs [44]. Experimental sepsis work also suggests that glutamine may alleviate immunosuppression by sustaining bacterial phagocytosis through mitochondrial mechanisms [45].

The adaptive immune compartment is similarly dependent on glutamine. T lymphocytes require glutamine for proliferation, cytokine synthesis, and maintenance of activation programs [39,40]. Although direct pediatric mechanistic evidence remains limited, disruption of this axis is biologically well positioned to contribute to both early inflammatory disequilibrium and later immune exhaustion in septic shock. In addition, extracellular glutamate may influence immune-cell activation through glutamate receptors and transporters, suggesting that the glutamate–glutamine axis may regulate inflammation through both intracellular metabolic pathways and extracellular signaling mechanisms [46].

### 3.2. Effects on Mitochondrial Function and Organ Energy Metabolism

The glutamate–glutamine axis is closely linked to mitochondrial integrity and organ energy metabolism, both of which are severely disturbed in septic shock. Under physiologic conditions, glutamine can be converted to glutamate and then to alpha-ketoglutarate, thereby replenishing tricarboxylic acid cycle intermediates and supporting oxidative phosphorylation [7,17]. During sepsis, however, mitochondrial respiration becomes inefficient, ATP generation declines, and organs shift toward maladaptive energy use, contributing to cellular dysfunction even when macrocirculation has been restored [34,47].

Experimental evidence supports a protective, context-dependent role of glutamine metabolism during mitochondrial stress. In oxidant injury models, glutamine preserves mitochondrial membrane potential, maintains oxygen consumption, and limits ATP depletion [41]. Sepsis-related studies further suggest that preservation of endogenous glutamine metabolism may attenuate endotoxin-induced injury through alpha-ketoglutarate-dependent mechanisms [48], while burn sepsis models indicate that glutamine supplementation can improve hepatic energy metabolism and reduce liver injury by stabilizing mitochondrial protein homeostasis [49]. These findings support a modifying or buffering role for the glutamate–glutamine axis, but they do not establish altered glutamine or glutamate as the dominant upstream mediator of mitochondrial dysfunction in pediatric septic shock.

These observations are especially relevant to tissues with high metabolic demand, including the heart, liver, skeletal muscle, and intestinal epithelium. Because the glutamate–glutamine axis supplies both carbon substrate for the tricarboxylic acid cycle and precursors for antioxidant defense, its disruption may intensify the bioenergetic crisis that underlies organ dysfunction in pediatric septic shock.

### 3.3. Effects on Intestinal Barrier, Gut–Liver Axis, and Disease Progression

The glutamate–glutamine axis is also closely linked to intestinal barrier integrity and gut–liver crosstalk, both of which are highly relevant to the progression of septic shock. Glutamine is a major fuel source for enterocytes and plays a key role in maintaining epithelial renewal, tight-junction stability, and mucosal immune defense [29,33]. When glutamine availability becomes insufficient during critical illness, the intestinal barrier may become vulnerable to epithelial injury, increased permeability, and microbial translocation [50].

The gut–liver axis has become an increasingly important concept in sepsis pathophysiology. Under physiologic conditions, the liver filters gut-derived microbial products and regulates inflammatory and metabolic responses to intestinal signals. During sepsis, however, gut barrier disruption and hepatic dysfunction can reinforce one another, creating a vicious cycle in which impaired barrier defense promotes translocation while liver injury reduces pathogen clearance and worsens inflammatory-metabolic instability [51,52].

Experimental evidence supports a protective role of glutamine in this context. Animal studies have shown that glutamine supplementation can reduce bacterial translocation and improve survival in models of gut-origin sepsis or impaired barrier function [53,54,55]. Although these findings cannot be directly extrapolated to clinical pediatric septic shock, they are biologically consistent with the known role of glutamine in epithelial integrity and mucosal redox balance.

## 4. Biomarker and Clinical Application Potential

### 4.1. Glutamine, Glutamate, and Related Ratios as Potential Biomarkers

Among metabolites linked to immunometabolic dysfunction in sepsis, glutamine and glutamate are of interest because they are biologically connected to immune-cell activity, mitochondrial substrate handling, redox balance, and epithelial barrier maintenance [7,34]. At present, however, their clinical relevance as biomarkers in pediatric septic shock remains insufficiently established. They should therefore be described as candidate research biomarkers rather than ready-to-use clinical tests. Their potential value lies in the possibility that they may provide information about metabolic stress that is not fully captured by conventional inflammatory or organ-injury markers, but this hypothesis requires pediatric-specific validation before clinical adoption.

The most direct candidate marker is plasma glutamine concentration. In critically ill populations, low glutamine at ICU admission has been associated with adverse outcomes, including higher mortality and greater illness severity [13,35]. Pediatric data remain limited, and available studies should be interpreted as hypothesis-generating rather than definitive evidence of clinical utility [35,36]. Mechanistically, low circulating glutamine may indicate a mismatch between systemic supply and the increased demands of immune cells, enterocytes, and injured organs during shock, but prospective pediatric validation is needed to determine whether this measurement adds actionable information beyond existing severity scores and biomarkers.

Glutamate is also a promising biomarker, although it has been less extensively studied than glutamine. Because glutamate is positioned downstream of glutaminolysis and upstream of alpha-ketoglutarate and glutathione synthesis, its concentration may reflect mitochondrial substrate handling and redox-related metabolic stress. Recent studies in sepsis and nutritional-risk populations suggest that glutamate-related metabolic patterns may carry independent prognostic information [56,57].

**Figure 2:**
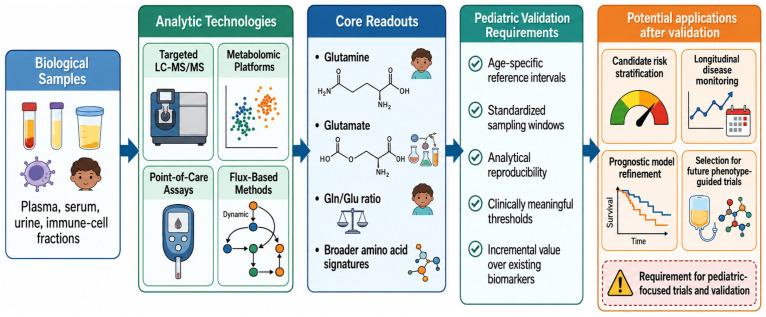
Proposed research-validation pathway for glutamate–glutamine axis biomarkers in pediatric septic shock.

Composite indices may ultimately be more informative than isolated metabolite levels, but this remains a research hypothesis. The glutamine-to-glutamate ratio is attractive because it may reflect the balance between substrate availability and metabolic consumption better than either analyte alone. More broadly, pediatric metabolomics studies show that multimetabolite signatures can distinguish septic shock and identify survival-associated metabolic patterns [9,11,12]. Even so, pediatric-specific reference ranges, decision thresholds, and incremental predictive value over established markers remain poorly defined, and circulating levels may not fully capture tissue-specific metabolism or temporal changes during shock progression, as shown in Figure 2.

This figure summarizes a proposed research-validation pathway rather than a currently validated clinical algorithm. Biological samples such as plasma, serum, urine, and immune-cell fractions may be analyzed using targeted LC-MS/MS, metabolomic platforms, point-of-care assays, or flux-based methods. Core readouts include glutamine, glutamate, the glutamine-to-glutamate ratio, and broader amino acid signatures. Before these readouts can be used for pediatric risk stratification, disease monitoring, prognostic assessment, or selection of children for metabolic or nutritional interventions, studies must establish age-specific reference intervals, sampling windows, analytical reproducibility, clinically meaningful thresholds, and incremental value over existing pediatric sepsis biomarkers.

### 4.2. Major Detection Methods and Technical Characteristics

The clinical evaluation of glutamine-, glutamate-, and pathway-related biomarkers depends heavily on the analytical platform used. At present, liquid chromatography coupled with tandem mass spectrometry is the most widely used and technically robust method for quantitative assessment of glutamine, glutamate, and related metabolites in plasma or serum. Compared with conventional biochemical assays, LC-MS/MS offers higher sensitivity, better specificity, and the ability to analyze multiple metabolites simultaneously [10,57,58,59].

For more detailed metabolic analysis, stable isotope-based methods provide information that static concentration measurements cannot capture. Stable isotope tracing combined with mass spectrometry allows investigators to follow labeled glutamine or related substrates through downstream pathways and thereby assess metabolic flux rather than simple abundance [60,61]. This is especially relevant for the glutamate–glutamine axis because septic shock is characterized by dynamic changes in substrate utilization, inter-organ redistribution, and tissue-specific reprogramming.

Point-of-care and biosensor-based strategies are also of interest because they may support faster bedside assessment. A bedside instrument for plasma glutamine screening in ICU patients has been validated against standard laboratory methods, suggesting that rapid clinical screening is technically feasible [62]. Earlier biosensor studies also demonstrated the feasibility of simultaneous glutamine and glutamate measurement in miniaturized platforms [63,64]. For pediatric septic shock, a tiered model may ultimately be most practical, with LC-MS/MS supporting biomarker validation and simplified bedside tools used for rapid risk assessment once clinically meaningful thresholds have been established.

### 4.3. Application Value in Early Identification, Disease Monitoring, and Prognostic Assessment

A potential advantage of biomarkers derived from the glutamate–glutamine axis is that they may capture metabolic stress that is not directly reflected by conventional markers of overt organ injury. Because glutamine and glutamate are involved in immune-cell activation, mitochondrial adaptation, and redox defense, changes in their levels could occur during early or evolving metabolic stress [7,34]. Pediatric metabolomics studies suggest that amino acid-related signatures can distinguish septic shock from control states and may be associated with mortality [9,11]. Nevertheless, these findings do not yet demonstrate that glutamine, glutamate, or their ratio are independently useful bedside biomarkers in pediatric septic shock.

These metabolites may also have value for longitudinal disease monitoring. Septic shock is a dynamic syndrome in which immune and metabolic states evolve over time, and a single measurement may not fully reflect disease trajectory. Studies of critically ill children have shown that glutamine concentrations and amino acid kinetics change during the course of severe sepsis and systemic inflammation [37]. Longitudinal metabolomics work in septic shock likewise demonstrates that serum metabolic profiles shift over the course of treatment and recovery [65].

The prognostic value of the glutamate–glutamine axis remains promising but incompletely validated. Low plasma glutamine at ICU admission has been associated with increased mortality or greater organ failure burden [13,35], while recent work suggests that abnormal glutamine and glutamate patterns may provide prognostic information in sepsis or nutritional-risk populations [56,57]. In pediatric biomarker research more broadly, the goal should not be to replace existing indicators but to test whether biologically meaningful metabolic markers improve risk prediction when added to clinical severity scores, lactate, inflammatory biomarkers, and organ dysfunction measures, as shown in Table 2 [12,66,67].

## 5. Therapeutic Implications and Future Perspectives

### 5.1. Nutritional and Metabolic Intervention Strategies Targeting the Glutamate–Glutamine Axis

Given the central role of the glutamate–glutamine axis in immune regulation, intestinal integrity, and mitochondrial adaptation, it is reasonable to consider this pathway as a therapeutic target in pediatric septic shock. The most direct strategy is glutamine supplementation, either as part of parenteral nutrition or enteral nutritional support. The rationale is biologically strong: during critical illness, endogenous glutamine production may become insufficient to meet the increased demands of immune cells, enterocytes, and injured organs, and restoration of glutamine availability could theoretically support host defense, preserve gut barrier function, and improve metabolic resilience [7,33,68].

However, clinical evidence remains mixed. Earlier systematic reviews suggested that parenteral glutamine supplementation might reduce hospital mortality or infectious complications in selected critically ill adults [69,70]. Later analyses of enteral supplementation showed no consistent overall benefit in unselected ICU populations, although some subgroup signals were observed [71]. More recent meta-analytic work continues to show uncertainty rather than clear consensus [72,73].

For pediatric septic shock, these issues are even more important because direct pediatric evidence is limited and adult data cannot simply be extrapolated to children. At present, glutamine-based intervention should not be regarded as an evidence-based precision nutritional therapy for pediatric septic shock. It is better framed as a hypothesis-generating strategy that requires careful prospective testing of nutritional risk, metabolic phenotype, timing, dose, route of administration, organ dysfunction, and safety. More broadly, the glutamate–glutamine axis may help guide future studies of integrated metabolic support, including optimized early enteral nutrition, preservation of gut barrier function, and interventions that stabilize mitochondrial and redox homeostasis, but these applications remain investigational, as shown in Table 3.

### 5.2. Current Limitations and Controversies

Despite growing interest in the glutamate–glutamine axis, several major limitations prevent its immediate clinical translation in pediatric septic shock. First, the current evidence base is still heavily weighted toward adult critical-care studies, experimental models, or mixed ICU populations. Direct pediatric septic shock data remain limited, and many available pediatric studies are observational or discovery-oriented rather than validation cohorts [10,12].

Second, interpretation of these biomarkers is complicated by biological and methodological heterogeneity. Plasma glutamine and glutamate levels are influenced by age, nutritional status, sampling time, organ dysfunction, and the dynamic stage of sepsis, while circulating concentrations may not accurately reflect intracellular metabolism or tissue-specific flux [37,57]. In addition, metabolomics studies differ in sample type, analytical platform, timing of collection, and statistical processing, which limits comparability across cohorts and reduces reproducibility [10,74,75].

A third controversy concerns intervention. Although the biological rationale for glutamine supplementation is strong, clinical trials and meta-analyses have produced inconsistent results, ranging from possible benefit to neutral outcomes and concerns about harm in specific critically ill populations [69,71,72,76]. This inconsistency suggests that the glutamate–glutamine axis is unlikely to represent a simple replace-the-missing-substrate problem. Instead, the clinical effect of intervention probably depends on metabolic phenotype, timing, dose, route of administration, and coexisting organ failure.

### 5.3. Future Perspectives and Clinical Outlook

Future research on the glutamate–glutamine axis in pediatric septic shock should move beyond descriptive metabolite measurement toward clinically actionable validation. The immediate priority is to determine whether glutamine-, glutamate-, and ratio-based indicators provide reproducible information that is independent of, and additive to, established clinical severity scores, lactate, inflammatory biomarkers, and organ dysfunction measures. This requires prospective multicenter pediatric cohorts with standardized sampling times, prespecified endpoints, and stratification by age, infection source, nutritional status, organ failure pattern, renal and hepatic function, and illness phase [77,78].

One important direction is incorporation of this axis into pediatric sepsis subphenotyping frameworks. Pediatric septic shock is increasingly recognized as a heterogeneous syndrome composed of biologically distinct subgroups rather than a single uniform disease entity [77,78,79]. In that context, glutamine- and glutamate-related biomarkers may be most valuable if they help identify children with marked metabolic stress, mitochondrial vulnerability, gut-barrier dysfunction, or nutritional risk. Such use would require predefined thresholds and external validation; otherwise, metabolite differences may remain mechanistically interesting but clinically non-actionable.

A second priority is methodological standardization. Studies should harmonize sample type, anticoagulant choice, processing delay, storage conditions, fasting or feeding status, analytical platform, calibration procedures, and statistical processing. Serial sampling should be emphasized because metabolic biomarkers are likely to be more informative when interpreted dynamically rather than as isolated baseline values [37,75]. Integration with single-cell immune profiling, transcriptomics, microbiome data, and machine learning-based risk prediction may further clarify how alterations in the glutamate–glutamine axis relate to evolving immune dysfunction and organ-specific injury [4,79].

A third priority concerns interventional research. Future trials should avoid unselected glutamine supplementation and instead test whether phenotype-guided approaches improve outcomes in clearly defined pediatric subgroups. Trial designs should incorporate nutritional baseline status, renal and hepatic function, shock phase, enteral tolerance, route and dose of supplementation, and safety monitoring. Until such evidence is available, the glutamate–glutamine axis should be viewed as a biologically plausible therapeutic target and a tool for hypothesis generation, not as a proven treatment pathway.

### 5.4. Limitations of This Narrative Review and the Underlying Evidence

This review has several limitations related to both its design and the underlying evidence base. As a narrative review, it used a transparent but non-systematic search and selection strategy and did not include PRISMA-style study flow reporting, quantitative synthesis, or pooled effect estimates. Although evidence was appraised qualitatively using risk-of-bias domains relevant to observational, interventional, and experimental studies, a formal study-by-study scoring table was not generated because the included literature spans heterogeneous clinical cohorts, animal models, cell-based studies, metabolomics discovery work, and prior reviews. Therefore, selection bias, publication bias, and uneven weighting of available evidence cannot be fully excluded.

The underlying studies also have important limitations. Direct pediatric septic shock data remain scarce, and many available pediatric studies are small, observational, single-center, or discovery-oriented. Sampling time, nutritional status, renal and hepatic dysfunction, vasoactive support, and illness phase may all influence glutamine and glutamate concentrations, but these variables are not consistently measured or adjusted for across studies. Adult ICU trials and experimental sepsis models provide useful biological context, yet they cannot be assumed to predict biomarker performance or therapeutic benefit in children. These limitations explain why the present review interprets the glutamate–glutamine axis as biologically plausible and translationally promising, but not yet clinically validated.

## 6. Conclusions

The glutamate–glutamine axis represents a biologically coherent framework for understanding pediatric septic shock, but its clinical relevance remains to be fully established. As a central hub linking immune-cell function, mitochondrial substrate handling, oxidative stress defense, and intestinal barrier integrity, this metabolic axis provides a plausible explanation for how systemic infection may contribute to persistent inflammation, organ dysfunction, and adverse outcomes in critically ill children [3,4,7,15,34].

From a translational perspective, glutamine, glutamate, and related metabolic indices should currently be regarded as candidate biomarkers rather than established clinical tools [9,11,12,57]. The strongest evidence supports biological plausibility and association with metabolic stress; the weakest evidence concerns pediatric-specific thresholds, incremental prognostic value, and intervention selection. Key barriers include the scarcity of pediatric validation studies, heterogeneity in metabolomic methodologies, lack of age-specific reference ranges, and unresolved controversy surrounding glutamine-based interventions [35,37,69,71,76].

Overall, the glutamate–glutamine axis should be viewed as a promising but still investigational bridge between mechanism and clinical application in pediatric septic shock. The conclusions of this review are therefore intentionally limited: the axis is mechanistically relevant, may help generate candidate biomarkers and phenotype-guided hypotheses, and warrants prospective pediatric validation before being used to guide clinical decisions. Future work should prioritize multicenter pediatric cohorts, longitudinal metabolic profiling, standardized analytical pipelines, explicit risk-of-bias assessment, and integration of metabolomics with immune phenotyping and precision medicine strategies [75,77,78,79].

## Figures and Tables

**Table 1:** Physiological functions and septic alterations of the glutamate–glutamine axis.

Component	Physiological Role	Alteration in Pediatric Septic Shock	Potential Consequence
Glutamine	Major circulating amino acid; supports immune-cell proliferation, enterocyte fuel use, nucleotide synthesis, and nitrogen transport	Relative depletion due to increased demand, altered redistribution, and insufficient endogenous supply	Immune dysfunction, impaired gut barrier maintenance, reduced metabolic reserve
Glutamate	Central metabolic intermediate linking amino acid turnover, alpha-ketoglutarate production, and glutathione synthesis	Altered turnover and compartmental imbalance during inflammatory stress	Mitochondrial substrate imbalance and impaired redox buffering
Glutaminase	Converts glutamine to glutamate and ammonia; initiates glutaminolysis	Upregulated or redistributed glutamine utilization under stress conditions	Accelerated substrate consumption and altered cellular fuel preference
Glutamine synthetase	Re-synthesizes glutamine and supports ammonia detoxification	May become insufficient relative to systemic demand during severe catabolism	Reduced glutamine replenishment and impaired adaptive compensation
Glutamate dehydrogenase and aminotransferases	Connect glutamate to the tricarboxylic acid cycle and inter-organ nitrogen exchange	Stress-related shifts in anaplerosis and nitrogen handling	Bioenergetic instability and organ-specific metabolic dysfunction
Glutathione pathway	Uses glutamate for antioxidant defense and redox homeostasis	Functional limitation when glutamine-glutamate flux is impaired	Oxidative stress, mitochondrial injury, and cellular damage

**Table 2:** Candidate biomarkers and detection strategies related to the glutamate–glutamine axis.

Biomarker or Index	Sample Type	Main Detection Method	Potential Research or Future Clinical Value	Major Limitation
Plasma glutamine	Plasma or serum	Targeted LC-MS/MS; point-of-care screening	Candidate marker for metabolic risk assessment and severity evaluation	Affected by timing, nutrition status, and non-sepsis critical illness
Plasma glutamate	Plasma or serum	Targeted LC-MS/MS	Candidate readout of downstream glutaminolysis and metabolic stress	Less validated in pediatric septic shock than glutamine
Glutamine-to-glutamate ratio	Plasma or serum	Calculated from targeted metabolite quantification	May reflect substrate-consumption balance better than single-analyte testing	Thresholds are not standardized in children
Multimetabolite amino acid panel	Plasma or serum	Targeted or untargeted metabolomics	May improve discrimination of disease severity and prognosis after validation	Higher cost and platform-related heterogeneity
Glutamine/glutamate flux measurements	Plasma with tracer studies	Stable isotope tracing plus mass spectrometry	Mechanistic assessment of turnover and metabolic reprogramming	Primarily a research tool with limited bedside feasibility

**Table 3:** Potential therapeutic strategies targeting the glutamate–glutamine axis in pediatric septic shock.

Strategy	Rationale	Hypothesized Benefit	Current Concern or Limitation
Glutamine supplementation	Restores substrate availability during relative depletion	May support immune function, gut barrier maintenance, and metabolic resilience in selected patients	Clinical benefit remains inconsistent; optimal timing and patient selection are unclear
Early enteral nutrition optimization	Maintains intestinal substrate delivery and mucosal integrity	May reduce barrier failure and gut-derived inflammatory amplification	Evidence is indirect and not specific to glutamine alone
Biomarker-informed nutritional stratification	Would target intervention to children with validated evidence of metabolic stress or nutritional risk	Could reduce indiscriminate supplementation if validated prospectively	Requires validated pediatric thresholds and prospective testing
Mitochondrial and redox support strategies	Stabilize energy production and antioxidant defense linked to glutamine metabolism	May limit organ dysfunction driven by oxidative and bioenergetic injury	Mechanisms are plausible, but pediatric interventional evidence is sparse
Integrated investigational metabolic therapy	Combines metabolomics, clinical phenotyping, and targeted support	May identify high-risk subgroups for individualized care in future studies	Currently conceptual and requires multicenter validation

## Data Availability

No new data were created or analyzed in this study. Data sharing is not applicable to this article.
